# Recovery-Oriented Education: The Impact of Visiting a Clubhouse on Psychiatry Residents

**DOI:** 10.1007/s40596-026-02363-3

**Published:** 2026-05-21

**Authors:** Joseph D. Guillory, Vishal J. Thakkar, Adriane M. dela Cruz

**Affiliations:** University of Texas Southwestern Medical Center, Dallas, TX USA

**Keywords:** Recovery-oriented care, Residency training, Education, Clubhouse

## Abstract

**Objective:**

Recovery‑oriented care emphasizes supporting individuals with serious mental illness in building meaningful, self-directed lives. This brief report describes a 4‑h, community‑based didactic held at a local clubhouse and evaluates its impact on psychiatry residents’ perceptions of recovery‑oriented care.

**Methods:**

Fifty-nine postgraduate year-2 psychiatry residents participated in a 4‑h didactic at a community clubhouse across two training years. Residents completed a 12-item survey assessing perceived importance of, training in, and comfort with recovery‑oriented care immediately before and after the session. An open-ended question gathered post-session feedback.

**Results:**

Residents’ agreement with all survey items increased significantly from pre‑ to post-didactic. The largest increases were observed in comfort discussing recovery-oriented care with patients, colleagues, and community organizations, and in comfort applying recovery principles in clinical practice. Qualitative responses most commonly reflected gratitude for the experience and emphasized the value of peer-led, community-based learning.

**Conclusion:**

A brief, clubhouse-based didactic was associated with increased recognition of the importance of recovery‑oriented care and greater comfort engaging with recovery principles. Community‑based, peer‑led educational experiences may be a valuable component of psychiatry residency training.

Recovery‑oriented care is a person-centered, strengths-based approach to the treatment of mental illness that emphasizes empowering individuals to live meaningful, self-directed lives defined by their own values, goals, and aspirations [1]. This approach represents a paradigm shift away from an exclusive focus on symptom reduction toward supporting recovery processes such as hope, empowerment, identity, meaning, and connectedness [2–4].

The clubhouse model is a community-based, recovery-oriented approach to psychosocial rehabilitation for individuals with serious mental illness and has been associated with reductions in psychiatric hospitalizations, improved employment outcomes, enhanced social connectedness, and improved quality of life [5]. These benefits have generated increasing interest in incorporating formal education on these concepts within psychiatry residency training programs [6].

This brief report describes an innovative approach to recovery-oriented care education in an adult psychiatry residency program through an annual, 4-h didactic session held at a local clubhouse. The study examines changes in resident perceived importance of, training in, and comfort with recovery-oriented care using pre- and post-didactic surveys. We hypothesized that residents’ ratings would increase from pre- to post-didactic.

## Methods

The authors conducted this study at a large academic medical center located in an urban setting in the southern United States. The recovery‑oriented didactic curriculum described here has been delivered annually as part of postgraduate year (PGY)−2 psychiatry didactics. Data for the present analysis were collected across two consecutive training years, during which 59 PGY-2 psychiatry residents participated in the same 4‑h clubhouse-based didactic session. The study was reviewed by the University of Texas Southwestern Medical Center Institutional Review Board and determined to qualify as non-regulated research.

Psychiatry residents completed a 12-item survey assessing perceptions of recovery‑oriented care immediately before (pre-didactic) and after (post-didactic) the 4-h session held at a community‑based clubhouse for adults with serious mental illness. Survey items were developed by the first and third authors and have not yet been formally validated. Responses were recorded on a five-point Likert scale (1 = strongly disagree; 5 = strongly agree). Surveys were administered using the Research Electronic Data Capture (REDCap) platform, with items presented on a single screen in the same order at both time points [7, 8]. The post-didactic survey also included one open-ended question inviting resident feedback.

The 12 Likert‑style survey questions (Table 1) were grouped a priori into three categories: perceived importance of recovery-oriented care (Question 1), perceived training in recovery-oriented care (Questions 2–8), and residents’ comfort engaging with recovery-oriented care in clinical and community contexts (Questions 9–12).

**Table 1:** Summary statistics from all survey questions presented before and after the PLAN didactic field trip (*N *= 59)

Survey question	Pre-didactic	Post-didactic	Change	Comparison
1. I believe that recovery-oriented education is important	3.64 (0.52)	3.88 (0.33)	+ 0.24	*t* = 3.90, *p* < 0.001, *d* = 0.51
2. I believe that recovery-oriented residency education should be community based	3.54 (0.68)	3.93 (0.25)	+ 0.39	*t* = 4.47, *p* < 0.001, *d* = 0.58
3. I believe that recovery-oriented residency education should be led by mental health peers	3.41 (0.65)	3.85 (0.36)	+ 0.44	*t* = 5.99, *p* < 0.001, *d* = 0.78
4. I believe that recovery-oriented residency education should be a component of my residency training	3.53 (0.60)	3.80 (0.41)	+ 0.27	*t* = 4.29, *p* < 0.001, *d* = 0.56
5. I believe that recovery-oriented residency education should be incorporated over all four years of my residency training	3.34 (0.76)	3.71 (0.67)	+ 0.37	*t* = 4.47, *p* < 0.001, *d* = 0.58
6. I believe that recovery-oriented residency education should be included in didactics	3.44 (0.65)	3.83 (0.38)	+ 0.39	*t* = 5.37, *p* < 0.001, *d* = 0.70
7. I believe that recovery-oriented residency education should be included in my clinical training	3.46 (0.68)	3.78 (0.49)	+ 0.32	*t* = 4.88, *p* < 0.001, *d* = 0.64
8. I have an interest in learning more about mental health recovery	3.54 (0.68)	3.80 (0.45)	+ 0.26	*t* = 3.09, *p* = 0.003, *d* = 0.40
9. I would be comfortable talking with patients about mental health recovery	2.61 (1.05)	3.70 (0.46)	+ 1.09	*t* = 8.29, *p* < 0.001, *d* = 1.08
10. I would be comfortable talking with professional colleagues about mental health recovery	2.49 (0.88)	3.66 (0.48)	+ 1.17	*t* = 11.05, *p* < 0.001, *d* = 1.44
11. I feel comfortable partnering with mental health recovery organizations	2.34 (0.88)	3.64 (0.55)	+ 1.30	*t* = 11.71, *p* < 0.001, *d* = 1.52
12. I feel comfortable applying mental health recovery concepts in my clinical practice	2.19 (0.96)	3.61 (0.67)	+ 1.42	*t* = 12.50, *p* < 0.001, *d* = 1.63

For each question, pre and post data are presented as mean (standard deviation). All 59 residents responded to all questions, so there was no missing data. All comparisons were significant at *p* < 0.01, and all comparisons survived the Bonferroni correction for 12 comparisons (new *p* < 0.0042)

The didactic was conducted at a community‑based clubhouse operating as a recovery‑oriented psychosocial rehabilitation program and does not provide residential or housing services. Core clubhouse staff and participating clubhouse members were largely consistent across both study years, with minor variation in participating members based on availability. The 4-h session was structured around established principles of mental health recovery and recovery‑oriented care and employed a constructivist learning framework [1, 9, 10]. The overall structure, sequencing, duration, and learning objectives of the session were maintained across both years to support curricular fidelity.

The session began with a 20-min faculty-led introductory presentation followed by a 45-min lunch-based orientation where residents and clubhouse members sat together to facilitate informal interaction. Next, a 45-min experiential segment was led by clubhouse members followed by a 30-min presentation by the clubhouse director on community therapy [11]. Clubhouse members then volunteered personal recovery narratives to facilitate 30 min of dialogue. The session concluded with 60 min of small-group case discussions involving residents and clubhouse members. See Table 2 for more session details.

**Table 2:** Structure and content of the clubhouse-based recovery-oriented didactic session

Didactic component	Time (min)	Facilitator(s)	Core content and educational approach
Introductory presentation	20	Faculty psychiatrist	Overview of recovery-oriented care; objectives of clubhouse visit; integration within longitudinal residency curriculum
Clubhouse orientation & lunch	45	Clubhouse staff and members	Introduction to clubhouse model, services, and daily operations; informal resident–member interaction
Wellness activity & clubhouse tour	45	Clubhouse members	Experiential wellness activity; tour of program spaces; discussion of recovery supports and participation
Community therapy presentation	30	Clubhouse director	Principles of community therapy and recovery-oriented psychiatric rehabilitation
Member recovery narratives	30	Clubhouse members	Personal recovery stories highlighting strengths, identity, meaning, and community
Small-group case discussions	60	Residents and clubhouse members	Composite clinical cases emphasizing collaboration, community integration, and recovery-oriented planning

Pre- and post-didactic survey responses were analyzed using JASP version 0.18.2.0. Twelve paired-samples *t* tests compared pre- and post-survey responses for each item. To account for multiple comparisons, a Bonferroni correction was applied, with statistical significance defined as *p* < 0.0042.

Qualitative responses to the open-ended question were analyzed using a thematic approach. The first and second authors independently reviewed all responses and assigned them to one or more thematic categories. Independent coding by the two authors demonstrated approximately 95% initial agreement, with the remaining discrepancies resolved through discussion until consensus was achieved.

## Results

All 59 residents completed pre-didactic and post-didactic surveys, so there was no missing data, and all residents’ data were included in the analyses. From pre-didactic to post-didactic, residents’ level of agreement to all survey questions significantly increased, with medium-to-very large effect sizes (*d*s > 0.40; Table 1).

Survey items were grouped a priori into three subscales: importance (Question 1), training (Questions 2–8), and comfort (Questions 9–12). Mean change scores differed across these categories. The importance subscale showed a modest increase (+ 0.24). The training subscale demonstrated small-to-moderate mean increases across items (range: + 0.26 to + 0.44). In contrast, the comfort subscale demonstrated the largest mean increases (range: + 1.09 to + 1.42), indicating substantially greater self-reported comfort discussing recovery-oriented care with patients and colleagues and applying recovery principles in practice. All paired-samples comparisons remained statistically significant following application of a Bonferroni correction for 12 comparisons (adjusted significance threshold p < 0.0042).

Thirty-seven residents (63%) provided responses to the post-session open-ended question. Qualitative responses most frequently reflected expressions of gratitude for the experience (67%), followed by comments highlighting the value of hearing personal recovery narratives from clubhouse members (20%) and learning about community‑based education and resources (33%). A smaller subset of residents provided specific feedback related to future training needs or referral pathways. These themes underscored the perceived value of peer‑led, community‑based learning.

## Discussion

This brief report describes the impact of a single, 4-h clubhouse-based didactic session on psychiatry residents’ perceptions of recovery-oriented care. While the didactic is offered annually, this evaluation reflects residents’ responses to a single educational exposure, assessed across two consecutive years in distinct PGY-2 cohorts.

Quantitative findings demonstrated statistically significant pre- to post-didactic increases across all survey categories, with the largest gains observed in residents’ comfort engaging in recovery‑oriented discussions and practices. This pattern is clinically meaningful, as recovery‑oriented care emphasizes collaboration, hope, identity, meaning, and community participation—domains that are often less explicitly addressed in traditional hospital-based psychiatric training. Empirical recovery‑oriented interventions, including peer support, supported employment, and community‑based rehabilitation models, have been associated with improved quality of life, functional outcomes, and self-efficacy among individuals with serious mental illness [11–13]. Experiencing these principles in a clubhouse setting, and learning directly from clubhouse members with lived experience, may have contributed to residents’ marked increases in comfort by illustrating how recovery‑oriented care is enacted beyond symptom-focused clinical encounters.

Improvements observed in the importance and training items suggest a growing interest among adult psychiatry residents who are exposed to recovery-oriented care education. Residents entered the session with relatively high baseline endorsement of these concepts, yet still demonstrated significant gains, indicating that experiential learning can deepen understanding beyond abstract agreement. Holding the session at a clubhouse likely played a critical role in this process by situating recovery concepts within an authentic community context and allowing residents to learn directly from clubhouse members who model recovery in daily practice.

Qualitative responses further underscored the value of peer-led, community-based learning, with residents most frequently expressing gratitude for the opportunity to hear directly from clubhouse members. Several residents highlighted how personal recovery narratives and exposure to local resources deepened their understanding of recovery-oriented care, with one noting that “every psychiatrist should be able to learn from these types of resources and the patients.” Collectively, these reflections suggest that experiential learning in a recovery community setting can complement traditional didactics by grounding recovery concepts in lived experience and enhancing residents’ confidence in engaging with recovery-oriented care.

This study has several limitations. Outcomes were measured immediately following the session, and long-term retention or behavioral change could not be assessed. The pre-/post-design may also be subject to response-shift bias, whereby residents’ understanding of recovery‑oriented care evolved during the didactic, influencing post-ratings. Ceiling effects associated with the 5-point Likert scale may further have limited detectable change, particularly for importance and training items, where smaller numeric increases likely reflect higher baseline endorsement and should be interpreted cautiously despite statistical significance.

This brief report suggests that a single, clubhouse-based, peer‑led didactic is associated with increased perceived importance of, training in, and comfort with recovery‑oriented care among psychiatry residents. Future work should examine the delivery of recovery‑oriented education across multiple residency programs and training years to better understand its generalizability and impact. Longitudinal studies evaluating knowledge retention, clinical application, and sustained engagement with recovery principles are needed to determine how community‑based educational experiences can be optimally integrated into psychiatry training curricula.

## Declarations

### Data Sharing Statement

Questions regarding the data used in this can be directed to the corresponding author by email.
